# Mouth puffing phenomena of patients with obstructive sleep apnea when mouth-taped: device’s efficacy confirmed with physical video observation

**DOI:** 10.1007/s11325-022-02588-0

**Published:** 2022-03-11

**Authors:** Je-Yang Jau, Terry B. J. Kuo, Lieber P. H. Li, Tien-Yu Chen, Chun-Ting Lai, Pin-Hsuan Huang, Cheryl C. H. Yang

**Affiliations:** 1 Faculty of Medicine, and Institute of Brain Science, National Yang-Ming Chiao-Tung University, No. 155, Sec. 2, Li-Nong St., Beitou, Taipei, 11221 Taiwan; 2 Sleep Research Center, National Yang Ming Chiao Tung University, Taipei, Taiwan; 3 Clinical Research Center, Taoyuan Psychiatric Center, Ministry of Health and Welfare, Taoyuan, Taiwan; 4 Department of Otolaryngology, Cheng Hsin General Hospital, No. 45, Cheng Hsin St., Beitou, Taipei, 11221 Taiwan; 5 Department of Medical Research, China Medical University Hospital, China Medical University, Taichung, Taiwan; 6 Department of Speech Language Pathology and Audiology, College of Health Technology, National Taipei University of Nursing and Health Sciences, Taipei, Taiwan; 7 Department of Psychiatry, Tri-Service General Hospital, School of Medicine, National Defense Medical Center, Taipei, Taiwan; 8 Department of Health Promotion and Health Education, National Taiwan Normal University, Taipei, Taiwan; 9 Department of Education and Research, Taipei City Hospital, Taipei, Taiwan; 10 Brain Research Center, National Yang Ming Chiao Tung University, Taipei, Taiwan

**Keywords:** OSA, Sleep disorder breathing, Mouth breathing, Mouth puffing, Breathing monitoring

## Abstract

**Purpose:**

This study aimed to design a device to monitor mouth puffing phenomena of patients with obstructive sleep apnea when mouth-taped and to employ video recording and computing algorithms to double-check and verify the efficacy of the device.

**Methods:**

A mouth puffing detector (MPD) was developed, and a video camera was set to record the patients’ mouth puffing phenomena in order to make ensure the data obtained from the device was appropriate and valid. Ten patients were recruited and had polysomnography. A program written in Python was used to investigate the efficacy of the program’s algorithms and the relationship between variables in polysomnography (sleep stage, apnea-hypopnea index or AHI, oxygen-related variables) and mouth puffing signals (MPSs). The video recording was used to validate the program. Bland–Altman plot, correlations, independent sample *t*-test, and ANOVA were analyzed by SPSS 24.0.

**Results:**

Patients were found to mouth puff when they sleep with their mouths taped. An MPD was able to detect the signals of mouth puffing. Mouth puffing signals were noted and categorized into four types of MPSs by our algorithms. MPSs were found to be significantly related to relative OSA indices. When all participants’ data were divided into minutes, intermittent mouth puffing (IMP) was found to be significantly different from non-mouth puffing in AHI, oxygen desaturation index (ODI), and time of oxygen saturation under 90% (T90) (AHI: 0.75 vs. 0.31; ODI: 0.75 vs. 0.30; T90: 5.52 vs. 1.25; *p* < 0.001). Participants with severe OSA showed a higher IMP percentage compared to participants with mild to moderate OSA and the control group (severe: 38%, mild-to-moderate: 65%, control: 95%; *p* < 0.001).

**Conclusions:**

This study established a simple way to detect mouth puffing phenomena when patients were mouth-taped during sleep, and the signals were classified into four types of MPSs. We propose that MPSs obtained from patients wearing the MPD can be used as a complement for clinicians to evaluate OSA.

**Supplementary Information:**

The online version contains supplementary material available at 10.1007/s11325-022-02588-0.

## Introduction

Obstructive sleep apnea (OSA), characterized by recurrent interruptions of breathing during sleep, is the most common form of sleep-disordered breathing [1]. Sleep apnea events lead to oxygen desaturation and an increase in the carbon dioxide level in the blood. These events may occur up to hundreds of times during one night’s sleep time depending on the severity of OSA [2]. OSA is highly prevalent and grossly underdiagnosed [3]. Approximately one in five adults has at least mild OSA, and one in 15 has moderate or severe OSA. It is estimated that over 85% of patients with clinically significant and treatable OSA have never been diagnosed [4]. Features include snoring, witnessed apneas, and sleepiness [1]. The apnea and hypopnea events from OSA bring about substantial harmful health consequences [5]. These acute physiological disruptions evolve into long-term sequelae, such as hypertension, cardiovascular morbidities [4], stroke, arrhythmia [6], decrements in cognitive function [7], decreased mood and quality of life [8], and premature death [9].

A study examining the relationship between mouth breathing and sleep showed that the proportion of mouth breathing increases with age, especially in men [10]. Breathing with the mouth open during sleep is a common symptom for patients with OSA and has been identified as a risk factor for OSA in recent years. Previous studies have shown that people breathing through their mouths might have a more elongated and narrower upper airway, increasing the pharyngeal resistance and collapsibility of the pharyngeal airway, thus negatively affecting the OSA severity [11–13]. In addition, mouth breathing has been related to hypoxia, and patients with OSA who have mouth breathing symptoms may have a relatively high chance of being hypoxemic [14]. Another study found a relationship between oral, nasal, or oro-nasal breathing and OSA. Patients with OSA spend more time breathing orally and oro-nasally than simple snorers, and the apnea/hypopnea index (AHI) is a major determinant of the time spent breathing orally and oro-nasally [15]. The upper airway resistance during sleep is significantly lower during nasal breathing than during oral breathing. Breast upheaval level is used to measure breathing rate, and sensors attached to the mouth or the nose are used to measure airflow. Some studies have utilized devices that require too many wires attached to patients for measuring the respiratory movements and airflow, causing difficulty for patients to sleep normally. Subjects in these studies have been tested or observed at sleeping centers or laboratories, but few devices have been developed to be used at home.

Mouth breathing is considered to be detrimental to health. Mouth taping is one of the common measures to avoid mouth breathing [16]. However, some studies have reported that OSA symptoms are not alleviated or may even get worse when some patients are mouth-taped during sleep. It is therefore important to find out why those patients’ OSA symptoms get worse when mouth-taped in sleep. However, some studies have found that symptoms deteriorate in one-third of patients with OSA after they are mouth-taped [17]. Our group of investigators has observed that drug induced sleep endoscopy, patients with OSA perform a mouth puffing phenomenon when they are asleep possibly indicating that these patients are trying to breathe through the mouth. Currently, there is no evice capable of measuring this mouth puffing phenomenon.

In this study, we develope a simple device that can be easily used at home to detect the phenomenon of mouth puffing when study subjects are mouth-taped before sleep. We hypothesized that an accelerometer is able to detect the mouth puffing phenomenon, and an algorithm is able to explain the phenomenon meaningfully. 

## Methods

### Participants and study process

Eighteen patients suspected of OSA, aged from 23 to 57 years, were recruited for study at O2 Win Dental Clinic [the clinic] in Taiwan. The inclusion criteria were patients with OSA-associated symptoms, such as snoring and daytime sleepiness. The exclusion criteria were patients with chronic diseases (e.g., psychiatric diseases, neurological disorders, diabetes, chronic renal diseases, cancers, and cardiovascular diseases), cigarette or alcohol addiction, and known sleep disorders. All participants provided written informed consent. The procedures used in this study were approved by the Human Research Committee of the National Yang-Ming University, Taipei, Taiwan (YM107083E), and the study was performed in conformity with the declaration of Helsinki.

**Fig. 1:**
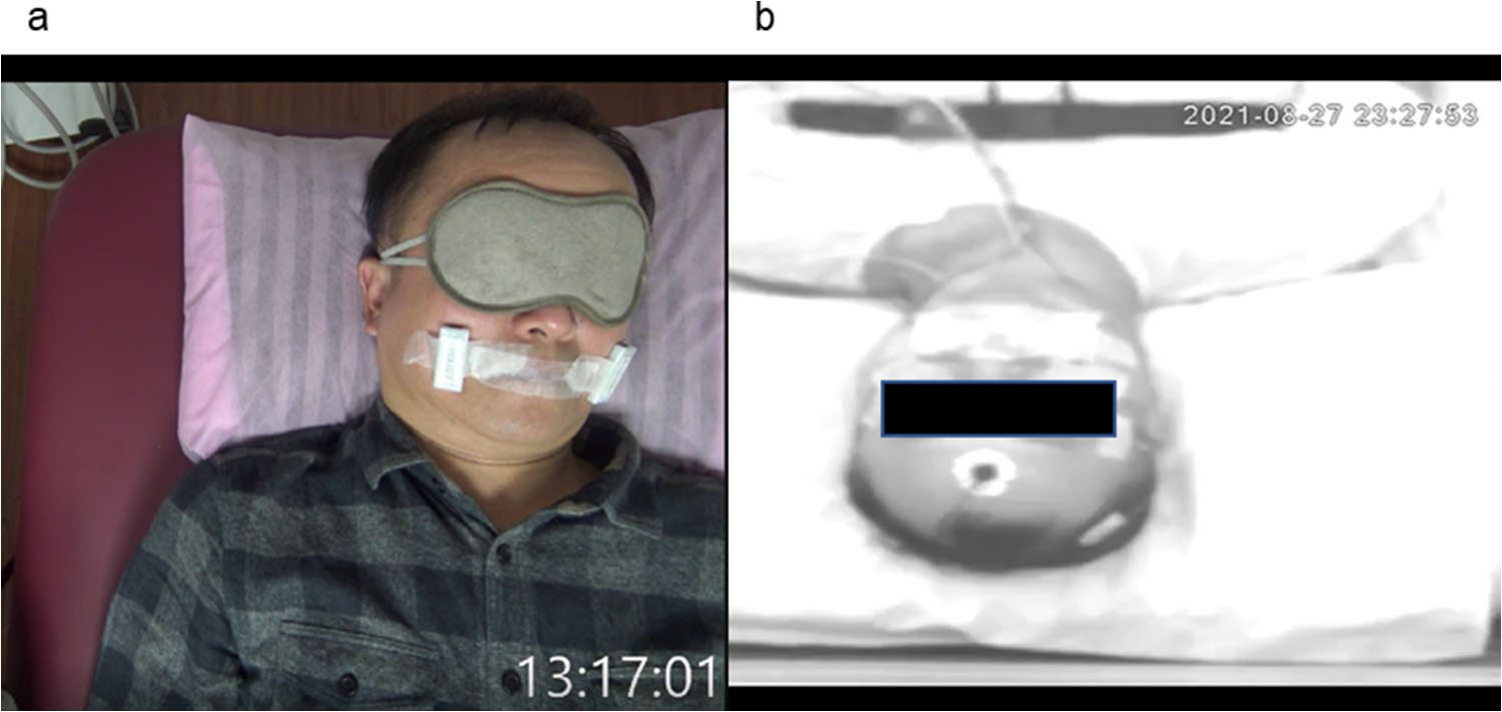
The images of the patient when video-recorded at the clinic (**a**) and at the laboratory (**b**)

All participants were observed at the clinic and their MPSs (signals obtained from the MPD) were obtained. These 18 walk-in participants self-reported that they had sleeping problems such as snoring, excessive daytime sleepiness, and bad quality of sleep at night. We used fingertip pulse oximetry for simple sleep testing. Other variables such as body mass index (BMI) and neck circumference were also measured. The patients, wearing a wireless fingertip pulse oximetry and a mouth puffing detector (MPD), were mouth-taped and tested at the clinic for 1–2 h, and their sleep was recorded by video placed above the bed looking down at the patients. The whole face of the patient was visible (Fig. 1a). After the testing trial, participants were asked to recall if they had fallen asleep and how long they have slept during the test. The participants were subsequently examined with polysomnography (PSG) with MPD and mouths taped for one night at National Yang-Ming University sleep laboratory (Fig. 1b). Questionnaires were completed by the participants. Sleep-related symptoms were evaluated using the Pittsburgh Sleep Quality Index (PSQI) and the Epworth Sleepiness Scale (ESS). The PSQI measures the quality and patterns of sleep; the ESS is an eight-item self-reported questionnaire that evaluates the level of daytime sleepiness as perceived by the patient. Again, we asked the participants to recall if they have fallen asleep and how long they have slept. The comfort level and tolerability of wearing PSG and MPD were also asked and reported.

### Instrumentation

#### Mouth puffing detector

**Fig. 2:**
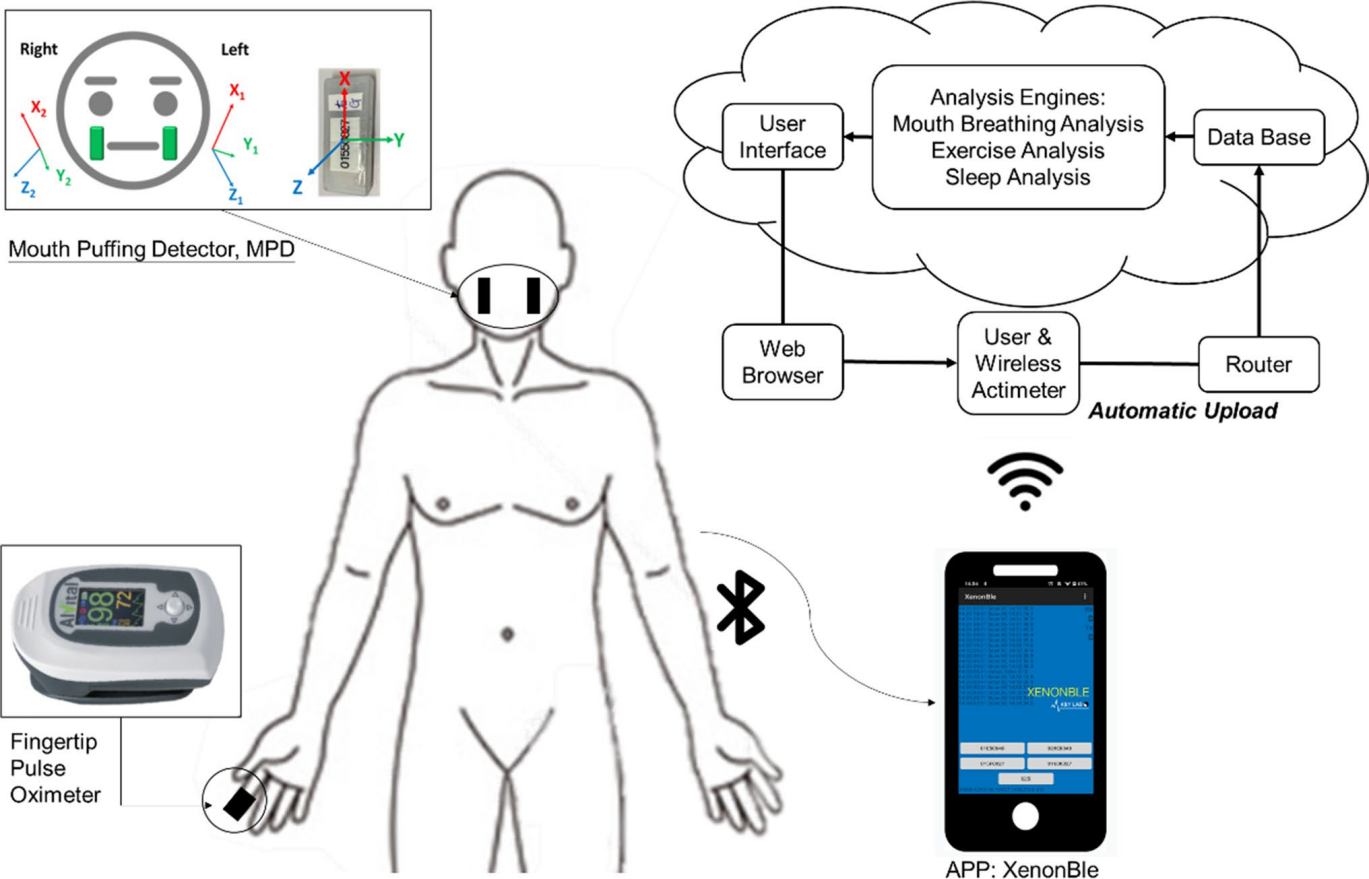
The diagram shows how the devices are utilized and data acquired. The participant, wearing a wireless fingertip pulse oximetry and an MPD, is mouth-taped and tested. All signals are instantaneously stored in a microcontroller and then intermittently transmitted to a router (mobile phone app, XenonBLE) with bluetooth. The router receives and relays the signals to a cloud server where the signals are processed and stored

To detect the phenomenon of puffing when mouth-taped, an MPD utilized two self-designed accelerometers (BLEACT, 7 g, 4.2 × 1.8 × 0.75 cm^3^, Taiwan) combined to record the ranges of mouth puffing. The ranges of mouth puffing were obtained from three axes, *x* (mediolateral), *y* (vertical), and *z* (anteroposterior), and within ranges from − 2 to + 2 g. Each axis had a sampling frequency of 125 Hz and was able to detect ranges from − 3 to 3 cm/s^2^ [18, 19]. The accelerometers were placed on both cheeks and fixed with tape (Fig. 2).

#### Fingertip pulse oximeter

A wireless fingertip pulse oximeter (AT101C-XB, Taiwan) is attached to a finger (Fig. 2), and the oxygen saturation signals (SpO_2_ signals) are uploaded to the server through a mobile phone and a bluetooth gateway. The oxygen saturation signal and physical activity data are gathered every second. The fingertip pulse oximeter used in the present study detects the oxygen saturation signal with an accelerometer for body movements detection, which further determines the total sleep time and reduces artifact interference. The device has been reported to have an 81% accuracy in OSA diagnosis [20]. The data gathered from all instruments are instantaneously stored in a microcontroller and then intermittently transmitted to a router via bluetooth. The router receives and relays all data to a cloud server where the data are stored and processed. The wireless transmitter power is < 1 µW and the wireless transmission range is approximately 10 m [18–20]. The percentage of total sleep time (TST) with oxygen saturation below 90% (T90) and the number of 3% or greater oxygen desaturations per hour (the oxygen desaturation index (ODI)) were assessed.

#### Overnight polysomnography

The overnight polysomnography (RESPIRONICS INC., USA) is performed by certified technicians to equip participants with a polysomnographic recorder in accordance with setup specifications [21] recommended by the 2007 American Academy of Sleep Medicine (AASM). PSG records electroencephalographic activity (EEG), electromylographic activity (EMG), electrocculogram (EOG), electrocardiograph (ECG), oxygen saturation (SaO2), the airflow, and the sleep position. The sampling rate is 200 Hz, and sleep stages are scored in 30-s epochs according to the 2007 AASM criteria. Apneas, hypopneas, and respiratory effort–related arousals are scored according to the 2012 AASM criteria [22].

### Signal processing

**Fig. 3:**
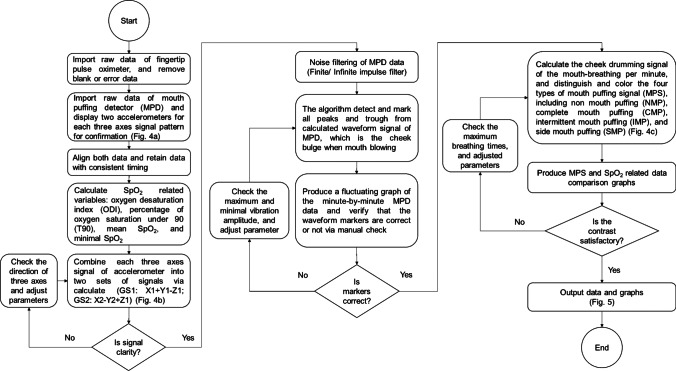
Diagram shows the process of device detection and data acquisition. The raw data from both devices were imported and blank and error data were removed. Two accelerometers were displayed for each of the three axes signal patterns per minute for confirmation (Fig. 4a) separately. Both data were aligned and retained with consistent timing. The SpO_2_-related variables were calculated. The data from the mouth puffing detector (MPD) were combined for each accelerometer into two sets of signals (GS1 (on the left): X1 + Y1-Z1; GS2 (on the right): X2-Y2 + Z2; Fig. 4b), and the signal clarity was assessed. If the signal was unclear, then the direction of each three axes signal and parameters were adjusted and checked. During noise filtering of the MPD data, the algorithms were detected, and all the peaks and troughs were marked from the calculated waveform signal of MPD, which is indicated by cheek bulge when mouth puffing. Then, a fluctuating graph of the minute-by-minute MPD data was produced, and the waveform markers were verified manually. If markers were mostly incorrect, then the vibration amplitude was checked, and the parameters were adjusted and calculated. The cheek drumming signal of the mouth breathing per minute was also calculated. Then, the four types of mouth breathing signal (MPS), including NMP (colored white), CMP (colored yellow), IMP (colored red), and SMP (colored green) were distinguished and colored (Fig. 4c). The MPS and SpO_2_-related data comparison graphs were produced (Fig. 5). If the contrast was unsatisfactory, then the maximum breathing times were checked, and the parameters were adjusted, followed by the final output data and graphs

**Fig. 4:**
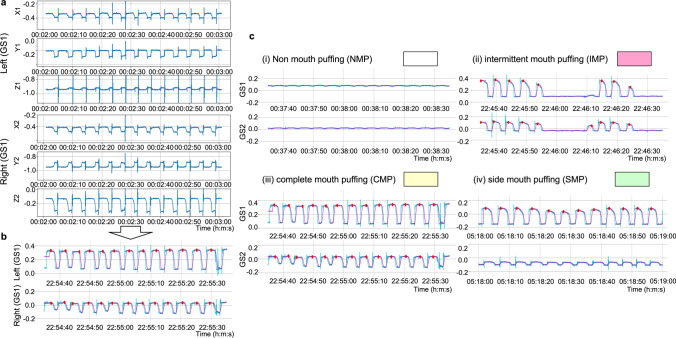
The processing of mouth puffing detector (MPD) data analysis. **a** Image of MPD data per minute with three axes in two accelerometers; **b** image of calculated MPD data per minute in the two accelerometers; **c** image of mouth breathing signal (MPS) meaning and color. To better understand the signal meaning, the MPD data per minute with three axes were split into two accelerometers by the algorithm (**a**) and the signal was calculated through its signal direction (**b**). Depending on the peak feature, there are four types of mouth breathing signals (**c**)

**Fig. 5:**
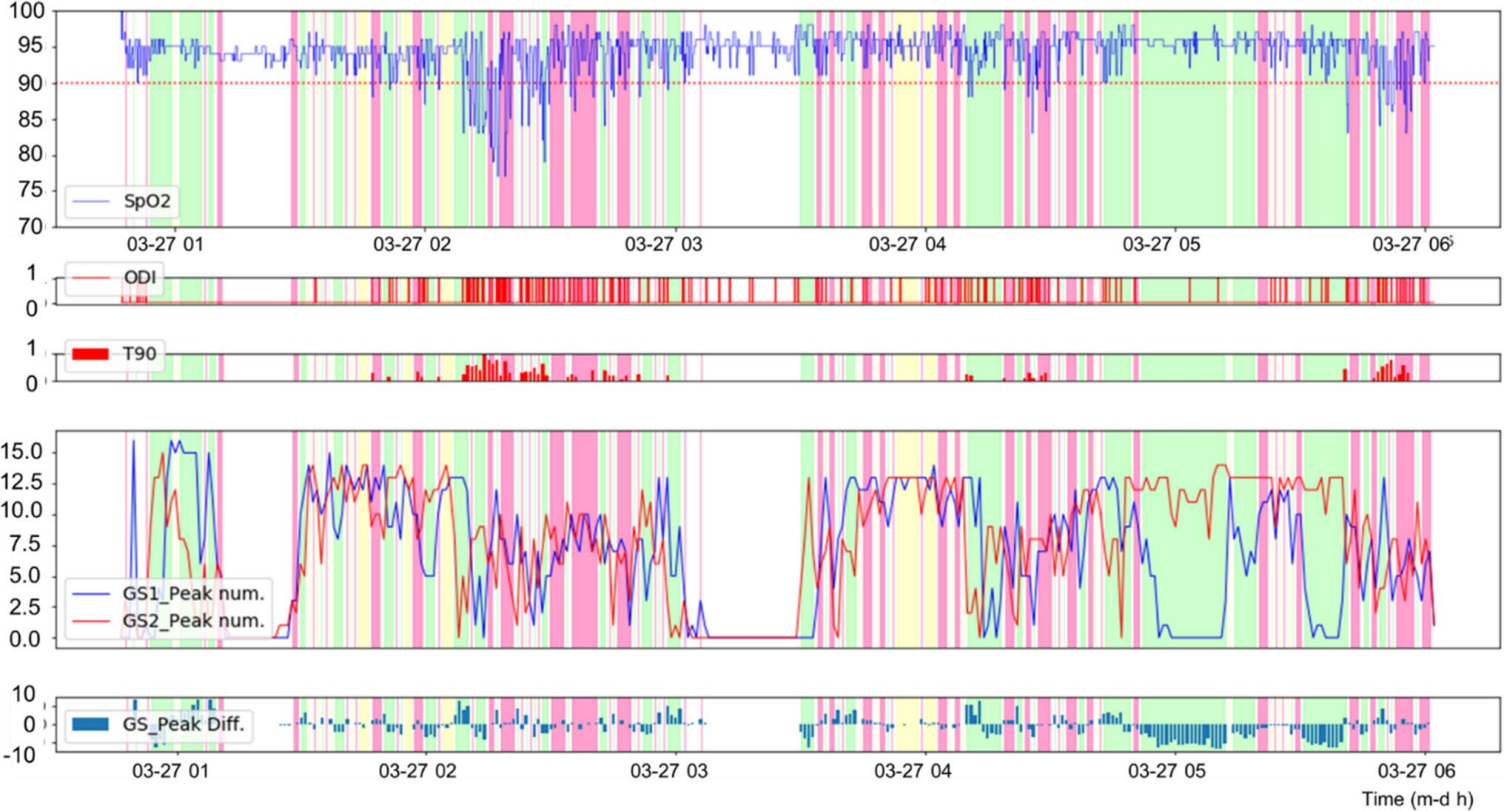
Image of the combined mouth puffing signal (MPS) and SpO2 variable data from one of the participants. After the analysis of MPS and SpO_2_, the MPS and SpO_2_ variable data were combined with color to understand the relationship between MPS and SpO_2_. For GS1 and GS2, the accelerometers are on the left and right sides, respectively; GS1 and GS2 peak num represents the number of waveform signals detected and marked by algorithms (GS1: X1 + Y1-Z1; GS2: X2-Y2 + Z2), and GS_Peak Diff. is the difference between GS1_Peak num and GS2_Peak num (GS_Peak Diff. = GS1_Peak num—GS2_Peak num)

The flowchart of the proposed algorithm is shown in Fig. 3. First, the raw data obtained from the fingertip pulse oximeter are imported from the PSG to be programmed. The raw data of the two accelerometers in the MPD are imported and displayed in a synchronized three-axis signal pattern (Fig. 4a). Only data of SpO_2_ and mouth puffing in the same time frame are retained for consistency. For the fingertip pulse oximeter data, the oxygen-related variables, including ODI (oxygen desaturation index is defined as a decrease in blood oxygen saturation to lower than 3% below the baseline) [23], T90 (percentage of oxygen saturation under 90% in total sleep time) [24], mean SpO_2_, and lowest SpO_2_ are calculated. For the data obtained from the MPD composed of two accelerometers (GS1: accelerometer on the left side, GS2: accelerometer on the right side), the data from each three-axis accelerometer are combined into a separate set of signals via calculation (GS1: X1 + Y1 + Z1; GS2: X2-Y2-Z2; Fig. 4b), and the signal clarity is checked. If the signal is unclear, the direction of each three-axis signal is checked, and the parameters are adjusted. The noise filtering of the MPD data (finite infinite impulse filter) is performed. The algorithms detect and mark all peaks and troughs from the calculated waveform signals of the MPD, which represent the extent of mouth puffing. Then, a fluctuating graph of the minute-by-minute MPD data is produced, and the waveform markers are verified manually. If the markers from a participant are mostly incorrect, then the maximum and minimum amplitude of vibration are checked and adjusted if necessary. The signals of puffing when mouth-taped are calculated per minute. Then, the mouth puffing signals (MPS) are distinguished and colored into four types, including non-mouth puffing (NMP, colored white), complete mouth puffing (CMB, colored yellow), intermittent mouth puffing (IMP, colored red), and side mouth puffing (SMP, colored green) (Fig. 4c). The comparison graphs of MPS- and SpO2-related data are shown in Fig. 5. If the contrast is unsatisfactory, then the maximum breathing times are checked and adjusted. The final output data and graphs are thus obtained.

### Statistical analysis

The statistical analysis is performed using SPSS software (24.0 vision for Windows). Independent sample *t*-test and ANOVA are used to compare the differences between different groups. Video recording, manual counts, and algorithmic counts of mouth puffing are compared with linear regression and Bland–Altman analysis. For the manual calculation part, we simply counted the waves in the figures exported from the collected MPD signals. We compared the results from manual MP calculation with the MP calculation by the designed algorithm to examine the accuracy of the algorithm. *P*-value < 0.05 is considered statistically significant.

## Results

Characteristics of the 18 participants (10 males; aged 23–57; mean age 43.0 years) were collected, including the BMI, the neck circumference, PSQI, and ESS (Table 1).

**Table 1:** Participants’ characteristics

Patient no	Gender	Age (years)	BMI (kg/m^2^)	Neck circumference (cm)	PSQI	ESS
1	M	46	25.6	41.5	10	10
2	M	46	23.5	41.2	9	13
3	M	39	30.1	39.8	11	10
4	M	47	31.3	46.3	8	13
5	M	36	37.4	46.6	10	16
6	F	49	33.3	38.5	12	14
7	M	54	26.4	45.0	7	8
8	M	54	29.1	43.4	6	9
9	M	57	22.8	37.3	5	12
10	M	36	27.1	39.7	12	19
11	M	52	26.1	38.5	7	10
12	M	30	36.3	44.7	11	14
13	M	51	22.4	37.9	6	8
14	M	51	21.6	33.0	5	9
15	F	32	21.6	31.0	4	2
16	F	23	23.2	28.5	6	3
17	F	40	16.4	28.0	4	2
18	F	30	31.1	33.0	3	2

*BMI*, body mass index; *PSQI*, Pittsburgh Sleep Quality Index; *ESS*, Epworth Sleepiness Scale

### MPD’s efficacy confirmed by physical video observation and the algorithm

**Fig. 6:**
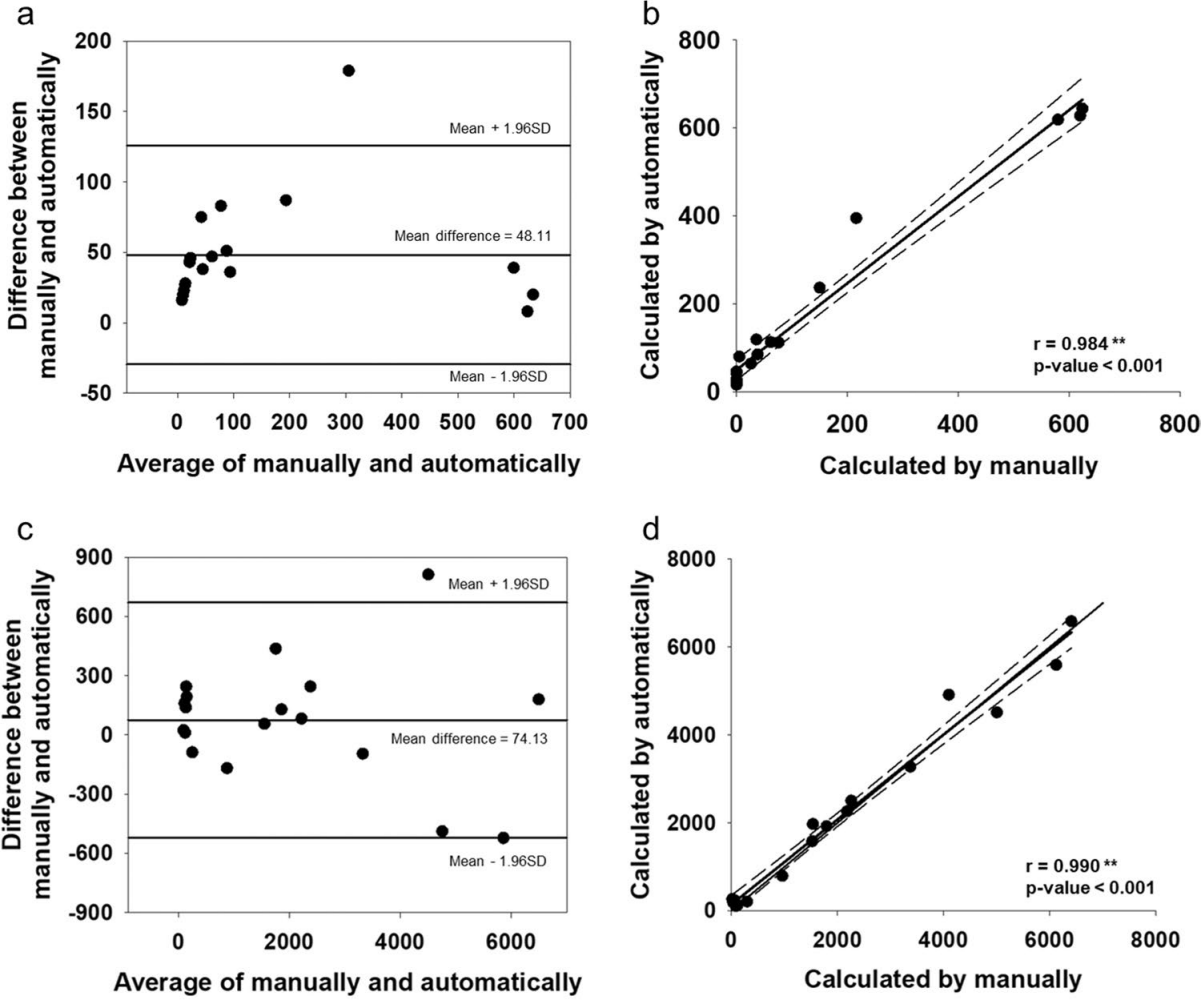
**a** Bland–Altman plot of the manual versus algorithm when participant slept in clinic; **b** correlation of mouth puffing signal (MPS) when participant slept in clinic; **c** Bland–Altman plot of the manual versus algorithm when participant slept with PSG in laboratory; **d** correlation of mouth puffing signal (MPS) calculated manually and automatically when participant slept with PSG in laboratory

Table 2 shows the data obtained at the clinic with a mean testing time of 77.9 min (ranging from 60–100 min). Observing from the video recording, we found 11 patients with the MP phenomenon and seven without. To verify the accuracy of our computer programming, Bland–Altman plot and correlation analysis were used to make sure the consistency between the marked waveforms generated by our algorithm and the expected waveforms calculated from human observation. As shown in Fig. 6, the MPSs of the 18 participants sleeping in clinic were calculated manually and automatically, more than 90% of the points on the Fig. 6a were within the 95% consistency boundary, and the average difference of all participants generated automatically and calculated manually was 48.11, indicating that some of the MPSs generated automatically underestimated the MPSs calculated manually. Figure 6b shows a positive correlation between the MPSs generated automatically and the MPSs calculated manually (*r* = 0.984, *p* < 0.001). Figure 6c and d shows the MPSs of the 18 participants sleeping in laboratory.

**Table 2:** Participants’ sleep data at clinic

Patient no	Sleep time (min)	Mean SpO2 (%)	Lowest SpO2 (%)	ODI (events/hour)	T90 (%)	NMP (%)	IMP (%)	CMP (%)	SMP (%)	Algorithm marked (times)	Video observed (times)
1	72	94.63	84.00	27.64	1.54	48.65	45.95	2.70	2.70	644	624
2	73	94.83	86.00	33.71	2.37	95.89	2.74	0	1.37	85	38
3	72	95.39	89.00	20.00	0.81	93.06	5.56	0	1.39	64	26
4	100	92.20	83.00	71.41	1.67	90.00	10.00	0	0	112	76
5	80	95.55	91.00	7.50	0	96.25	3.75	0	0	119	36
6	70	96.10	89.00	14.40	0.59	80.28	19.72	0	0	113	62
7	90	95.82	90.00	6.53	0.02	65.22	18.48	1.09	15.22	237	150
8	60	94.39	87.00	31.81	2.20	28.57	51.43	0	20.00	395	216
9	70	96.09	82.00	15.87	1.12	91.77	7.87	0	0	27	0
10	75	96.22	66.00	15.00	1.67	90.58	9.42	0	0	46	0
11	80	93.05	67.00	14.51	4.67	51.65	32.97	7.69	7.69	619	580
12	60	92.06	52.00	75.17	30.42	13.21	73.58	0	13.21	628	620
13	75	94.12	87.00	22.00	2.54	96.67	3.33	0	0	23	0
14	100	97.44	92.00	1.19	0.02	96.04	3.96	0	0	80	5
15	85	98.01	73.00	2.13	0.37	97.18	2.82	0	0	20	0
16	86	98.62	94.00	1.40	0.04	96.51	3.49	0	0	28	0
17	80	99.29	95.00	0.75	0.08	98.75	1.25	0	0	43	0
18	75	98.80	91.00	0.79	0	98.68	0	0	1.32	16	0

*ODI*, oxygen desaturation index; *T90*, percentage of oxygen saturation under 90; *NMP*, non-mouth puffing; *IMP*, intermit-tent mouth puffing; *CMP*, complete mouth puffing; *SMP*, side mouth puffing; algorithm marked means mouth puffing signal numbers by algorithm marked; video observed means mouth puffing signal numbers by video observed

### Sleep data obtained by PSG

A total of 18 participant’s sleep data were examined by PSG. The total sleep time of all 18 participants was 5997 min, i.e., a mean sleep time of 5.5 h for each participant. In order to understand the relationship between the changes in SpO_2_ and breathing patterns, we divided the data of all the participants by minutes. The four types of MPSs were analyzed by ANOVA and Scheffe’s post hoc tests were used. The mean AHI by minutes was found to be significantly different at the four mouth puffing signals, as were the mean OA by minutes, the mean ODI by minutes, the time of oxygen saturation under 90%, the mean SpO_2_, and the mean times of snoring by minutes (Table 3). Also, the mean AHI, OA, HYPO, ODI, and T90 values at IMP were found to be higher than those at NMP.

**Table 3:** Minute by minute AHI and other oxygen-related variables data’s differences at four initial MPSs

*N* = 5997	SMP (a) (*n* = 854)	CMP(b) (*n* = 114)	NMP(c) (*n* = 3661)	IMP(d) (*n* = 1368)	*P*-value
AHI(events/min)	0.69 ± 0.75	0.52 ± 0.72	0.31 ± 0.59^ab^	0.75 ± 0.75^bc^	< .001**
OA(events/min)	0.21 ± 0.49	0.19 ± 0.50	0.07 ± 0.31^ab^	0.34 ± 0.58^abc^	< .001**
CA(events/min)	0.01 ± 0.11	0.01 ± 0.09	0 ± 0.06	0.01 ± 0.11^c^	.007*
MA(events/min)	0.01 ± 0.11	0 ± 0	0 ± 0.04^a^	0 ± 0.06^a^	< .001**
HYPO(events/min)	0.46 ± 0.66	0.32 ± 0.55	0.23 ± 0.51^a^	0.40 ± 0.63^c^	< .001**
ODI(events/min)	0.66 ± 0.76	0.46 ± 0.64^a^	0.30 ± 0.58^ab^	0.75 ± 0.76^abc^	< .001**
T90 (%)	6.18 ± 13.73	3.10 ± 9.53^a^	1.25 ± 6.28^a^	5.52 ± 12.96^ac^	< .001**
Mean SpO_2_ (%)	93.87 ± 3.58	93.99 ± 2.41	96.32 ± 2.71^ab^	94.35 ± 3.47^ac^	< .001**
Snore (events/min)	6.52 ± 4.70	6.35 ± 4.98	2.83 ± 4.37^ab^	5.23 ± 4.50^ac^	< .001**

Analysis of variance and Scheffe’s post hoc tests are used. a = compared with side mouth puffing sleep (SMP), b = compared with CMP, c = compared with NMP; *p < 0.05, **p < 0.001. *AHI*, apnea/hypopnea index; *OA*, obstruct apnea; *CA*, centra apnea; *MA*, mix apnea; *HYPO*, hypopnea; *ODI*, oxygen desaturation index; *T90*, percentage of oxygen saturation under 90; *SMP*, side mouth puffing; *CMP*, complete mouth puffing; *NMP*, non-mouth puffing; *IMP*, intermittent mouth puffing; *n*, the number of data minutes

### Relationship between three groups’ OSA severity and oxygen-related variables and MPSs

All participants were classified into three different OSA severity groups based on their AHIs (normal or control group: AHI < 5 times/hour; mild-to-moderate OSA: 5 AHI < 30 times/hour; severe OSA: AHI 30 or more times/hour). In Table 4, data are presented from application of ANOVA and Scheffe’s post hoc tests.

**Table 4:** Relationships between three groups’ OSA severity and their oxygen-related variables and MPSs

	Normal (a) (*n* = 5)	Mild-to-moderate OSA (b) (*n* = 5)	Severe OSA (c) (*n* = 8)	*P-*value
Age (years)	35.2 ± 10.7	52.0 ± 4.1 ^a^	42.1 ± 10.0	.017*
BMI (kg/m^2^)	22.8 ± 5.3	24.7 ± 1.9	31.0 ± 4.6 ^a^	.009*
Neck circumference (cm)	30.7 ± 2.4	40.0 ± 3.2 ^a^	42.5 ± 3.2 ^a^	< .001**
ODI (events/hour)	2.1 ± 1.4	16.8 ± 7.3	57.6 ± 21.4 ^ab^	< .001**
T90 (%)	0.40 ± 0.75	1.64 ± 1.29	11.57 ± 16.14	.164
Mean SpO_2_ (%)	98.3 ± 0.4	95.5 ± 1.2	94.1 ± 2.8 ^a^	.008*
Lowest SpO_2_ (%)	87.6 ± 8.4	80.2 ± 7.7	66.9 ± 15.6 ^a^	.024*
Snore index (events/hour)	234.0 ± 366.3	1318.6 ± 1316.7	2320.1 ± 1118.6 ^a^	.011*
NMP (%)	95.28 ± 1.21	65.32 ± 14.91 ^a^	40.66 ± 18.78 ^ab^	< .001**
IMP (%)	3.65 ± 0.78	22.05 ± 6.57 ^a^	38.23 ± 12.15 ^ab^	< .001**
CMP (%)	0.17 ± 0.27	2.21 ± 2.45	1.99 ± 2.08	.197
SMP (%)	0.90 ± 0.83	10.41 ± 10.26	19.12 ± 17.02	.072

Analysis of variance and Scheffe’s post hoc tests are used. Normal: AHI < 5 events/hour (control group), mild-to-moderate OSA: 5≦AHI < 30; severe OSA: AHI≧30; a = compared with normal, b = compared with mild to moderate; *p < 0.05, **p < 0.001. *AHI*, apnea/hypopnea index; BMI, body mass index; *ODI*, oxygen desaturation index; T90, percentage of oxygen saturation under 90; *NMP*, non-mouth puffing; *IMP*, intermittent mouth puffing; *CMP*, complete mouth puffing; *SMP*, side mouth puffing

## Discussion

Mouth puffing, a derivation of mouth breathing, may possibly be a factor leading to OSA. Since the methodology of measuring mouth puffing is straightforward and easily incorporated into an analytic computer system, the mouth puffing phenomenon may be applied to future physiological research on sleep-related illness. In our study, several insights were revealed: (1) when mouth-taped, some patients with OSA show the symptom of mouth puffing, which can be detected with a MPD, and (2) the relationship between MPSs and both AHI and oxygen-related variables are shown by PSG data.

We have shown that the MP phenomenon does exist, that the MPD is able to identify the MP phenomenon, and that the MP signals can be marked by our algorithm. To verify the efficacy of the MPD, we found a high consistency between the physical observation and the algorithm (in laboratory: correlation = 0.990, *p* < 0.001), which demonstrated that our algorithm is effective in marking the MPSs. Though MPSs observed from the video recording and MPSs marked by the algorithm showed high correlation, the algorithm showed several kinds of aberrant signals like turning over, coughing, and tooth-grinding, which can be observed from the video recording. In order to identify the MPSs more accurately and to obliterate irrelevant signals, it will be necessary to modify the algorithm. Using a half-minute as the reporting unit, the waves were easily disrupted and the error rate was high. Using a minute as the reporting unit, it was possible to get 10–20 waves at a time, and the error rate was found to be an acceptable 5%. Using the MPSs recorded per minute allowed for correlations among sleep stages, AHI scores, and blood oxygen levels. In our experience, using a minute as the reporting interval is a feasible way of showing the data.

Mouth taping can prevent patients with OSA from inhaling with the mouth but cannot prevent patients from exhaling with the mouth. Many past studies have researched the relationship between oral/nasal breathing and OSA. The relationship between the mouth puffing phenomenon and OSA has now been evaluated for the first time in this study. Similar findings have been reported in other studies that patients who have a higher percentage of oral and oro-nasal breathing periods have more serious OSA and lower SpO2 than common snorers or healthy subjects [10, 12, 13, 15, 25]. However, our findings also indicate that mouth breathing should be divided into two categories, IMP and CMP, defined on a minute by minute basis. Differences among the four MPSs were further investigated. Examination shows that the IMP ratio is positively correlated with the ODI/T90 and negatively correlated with the mean SpO2, when compared with the other MPSs. Participants when completely breathing with nostrils (NMP) tend to have more stable SpO2 during sleep [12, 13, 15]. Though a regular breathing, CMP is positively correlated with lower SpO2 than NMP is, which indicates that CMP is a worse breathing pattern than NMP is.

Oral breathing is a common phenomenon of patients with OSA patients during sleep, happening more frequently right before and after events of apnea and hypopnea. An event of apnea or hypopnea is usually accompanied by a deep and long oral breathe, most likely because the patient tries to make up for oxygen depletion. Oral breathing, accompanied with other factors, causes events of apnea and hypopnea, which in turn causes oral breathing. It is a vicious cycle that oral breathing and events of apnea and hypopnea reinforce each other [15]. Past studies have shown that open mouth breathing tends to cause airway collapse. Mouth breathing patients with OSA tend to have more serious OSA and worse oximetric variables. Also, mouth breathing is associated with more serious and more prevalent lateral pharyngeal wall collapse and tongue base collapse [13].

We theorize that the mouth puffing phenomenon may be an indicator of OSA and may be useful in the diagnosis of OSA. In past studies, a face mask seal has commonly been used to detect airway flow to determine when nasal breathing or oral breathing is present. In the current study, we used the mouth puffing phenomenon to determine when nasal breathing or oral breathing was present and found the same relationship between oral breathing and OSA as other studies have. Similar findings have been reported in other studies that patients who have a higher percentage of oral and oro-nasal breathing periods have more serious OSA and lower SpO_2_ than common snorers or healthy subjects [11–13, 15].

The pathophysiological mechanism is that oral breathing ensues from upper airway resistance, which includes allergic nostril obstruction and constriction of the upper airway caused by bad habitual breathing habits [11, 12]. In our study, patient who used mount breathing tended to have a higher standard deviation of SpO_2_, a lower mean SpO_2_, and a lower T90 during sleep. A plausible explanation for the phenomenon is that when the patient breathes with the mouth, the ODI drops or fluctuates, which engenders a higher standard deviation of SpO_2_, a lower mean SpO_2_, and a higher T90 during sleep. After a period of apnea a big mouth breathe often occurs as if the patient is trying to catch up after being deprived of oxygen [10, 26].

In supplement materials, we also show that patients with OSA have worse AHI and ODI and higher percentage of IMP during stage REM sleep. In our study, three of ten patients with OSAs had higher AHI, ODI, and IMP during NREM sleep. We suggest that data obtained during stage REM sleep and during NREM be analyzed separately in future studies as we do in this study to avoid data from being skewed. Generally, people breathe more regularly during the NREM sleep and more irregularly during stage REM sleep. People breathe more irregularly during the REM sleep because muscles of pharynx slacken, are slow to inhibit apnea and hypopnea, and cause apnea and hypopnea to occur more frequently and longer. It is found that the geniogiossus muscle becomes very inactive during stage REM sleep, causes the tongue to slide back, causes the airway to be obstructed, and induces apnea and hypopnea. Prior studies have found that 50% of patients with OSA patients belong to the NREM-AHI group [28].

There are several limitations to our study. First, there were a low number of subjects recruited in our study. Second, the data were obtained at the clinic and laboratory, not the patient’s usual sleeping location and time, which may affect the data obtained. Third, this study used a cross-sectional design that suggests only correlations between MPSs and OSA and cannot infer causality.

## Conclusions

Our study supports the observation that the mouth puffing phenomenon during sleep when mouth-taped and mouth breathing during sleep are highly correlated. Future studies should investigate (1) whether or not all patients with OSA perform the mouth puffing phenomenon after being mouth-taped, (2) the correlation between the proportion of mouth puffing during sleep and OSA severity, and (3) which population groups are more likely to mouth puff while asleep. We expect that MPD may be useful to identify patients with OSA more easily and prompt them to undergo definitive evaluation and management.

## Supplementary Information

Below is the link to the electronic supplementary material.

Supplementary file1 (DOCX 35 KB)

## Data Availability

The datasets generated during and/or analyzed during the current study are available from the corresponding author on reasonable request.

## Declarations

### Patents

M610717: Sleep-Disordered-Breathing Screening Equipment.

## References

[1] Jordan AS, McSharry DG, Malhotra A (2014). Adult obstructive sleep apnoea. Lancet.

[2] Deegan PC, McNicholas WT (1995). Pathophysiology of obstructive sleep apnoea. Eur Respir J.

[3] Foldvary-Schaefer NR, Waters TE (2017). Sleep-disordered breathing. Continuum (Minneap Minn).

[4] Gonzaga C, Bertolami A, Bertolami M, Amodeo C, Calhoun D (2015). Obstructive sleep apnea, hypertension and cardiovascular diseases. J Hum Hypertens.

[5] Peppard PE, Young T, Barnet JH, Palta M, Hagen EW, Hla KM (2013). Increased prevalence of sleep-disordered breathing in adults. Am J Epidemiol.

[6] Salman LA, Shulman R, Cohen JB (2020). Obstructive sleep apnea, hypertension, and cardiovascular risk: epidemiology, pathophysiology, and management. Curr Cardiol Rep.

[7] Yaffe K, Laffan AM, Harrison SL (2011). Sleep-disordered breathing, hypoxia, and risk of mild cognitive impairment and dementia in older women. JAMA.

[8] Baldwin CM, Griffith KA, Nieto FJ, O'Connor GT, Walsleben JA, Redline S (2001). The association of sleep-disordered breathing and sleep symptoms with quality of life in the Sleep Heart Health Study. Sleep.

[9] Young T, Finn L, Peppard PE (2008). Sleep disordered breathing and mortality: eighteen-year follow-up of the Wisconsin sleep cohort. Sleep.

[10] Gleeson K, Zwillich CW, Braier K, White DP (1986). Breathing route during sleep. Am Rev Respir Dis.

[11] Kim EJ, Choi JH, Kim KW (2011). The impacts of open-mouth breathing on upper airway space in obstructive sleep apnea: 3-D MDCT analysis. Eur Arch Otorhinolaryngol.

[12] Fitzpatrick MF, McLean H, Urton AM, Tan A, O'Donnell D, Driver HS (2003). Effect of nasal or oral breathing route on upper airway resistance during sleep. Eur Respir J.

[13] Hsu YB, Lan MY, Huang YC, Kao MC, Lan MC (2021). Association between breathing route, oxygen desaturation, and upper airway morphology. Laryngoscope.

[14] Niaki EA, Chalipa J, Taghipoor E (2010). Evaluation of oxygen saturation by pulse-oximetry in mouth breathing patients. Acta Med Iran.

[15] Koutsourelakis I, Vagiakis E, Roussos C, Zakynthinos S (2006). Obstructive sleep apnoea and oral breathing in patients free of nasal obstruction. Eur Respir J.

[16] Stupak HD (2020) Strategies for addressing mouth-breathing treatment with an “adequate” nose. Rethink Rhinoplasty Facial Surg:193–207 10.1007/978-3-030-44674-1_9

[17] Huang TW, Young TH (2015). Novel porous oral patches for patients with mild obstructive sleep apnea and mouth breathing: a pilot study. Otolaryngol Head Neck Surg.

[18] Hsieh IT, Chen CY, Lin YC, Li JY, Lai CT, Kuo TB Application of cloud computing in physical activity research. In: 11th IEEE SENSORS 2012 Conference, 2012. p 6411560

[19] Kuo TBJ, Li JY, Chen CY (2018). Influence of accelerometer placement and/or heart rate on energy expenditure prediction during uphill exercise. J Mot Behav.

[20] Wu C-H, Lee J-H, Kuo TBJ, Lai C-T, Li LPH, Yang CCH (2020). Improving the diagnostic ability of the sleep apnea screening system based on oximetry by using physical activity data. J Med Biol Eng.

[21] Collop NA, Anderson WM, Boehlecke B (2007). Clinical guidelines for the use of unattended portable monitors in the diagnosis of obstructive sleep apnea in adult patients. Portable Monitoring Task Force of the American Academy of Sleep Medicine. J Clin Sleep Med.

[22] Yamakoshi S, Kasai T, Tomita Y (2016). Comparison of clinical features and polysomnographic findings between men and women with sleep apnea. J Thorac Dis.

[23] Gyulay S, Olson LG, Hensley MJ, King MT, Allen KM, Saunders NA (1993). A comparison of clinical assessment and home oximetry in the diagnosis of obstructive sleep apnea. Am Rev Respir Dis.

[24] Rey de Castro J, Huamaní C, Escobar-Córdoba F, Liendo C (2015). Clinical factors associated with extreme sleep apnoea [AHI>100 events per hour] in Peruvian patients: a case-control study-a preliminary report. Sleep Sci.

[25] Tsuda H, Lowe AA, Chen H, Fleetham JA, Ayas NT, Almeida FR (2011). The relationship between mouth opening and sleep stage-related sleep disordered breathing. J Clin Sleep Med.

[26] McKeown P, O'Connor-Reina C, Plaza G (2021). Breathing re-education and phenotypes of sleep apnea: a review. J Clin Med.

[27] McSharry DG, Saboisky JP, Deyoung P (2014). Physiological mechanisms of upper airway hypotonia during REM sleep. Sleep.

[28] Siddiqui F, Walters AS, Goldstein D, Lahey M, Desai H (2006). Half of patients with obstructive sleep apnea have a higher NREM AHI than REM AHI. Sleep Med.

