# Supplementary material for: Mouth puffing phenomena of patients with obstructive sleep apnea when mouth-taped: device’s efficacy confirmed with physical video observation
Source: Sleep Breath. 2022 Mar 11;27(1):153–64. doi: 10.1007/s11325-022-02588-0 (PMC9992075; doi:10.1007/s11325-022-02588-0)
Supplement: Supplementary file 1 — Supplementary file1 (DOCX 35 KB) [file 11325_2022_2588_MOESM1_ESM.docx]

**Supplementary Material**

In Table S-1, sleep stages are divided into non rapid eye movements (NREM, including N1, N2 and N3) and rapid eye movements. By independent sample t-test, the mean AHI, the mean ODI and the mean T90 are found to be statistically significantly higher and the mean SpO2 is found statistically significantly to be lower at REM stage than at NREM stages.

**Table S-1.** The differences of AHI, oxygen related variables, and MPSs at NREM and REM

|  | NREM (n=2939) | REM (n=766) | P-value |
| --- | --- | --- | --- |
| AHI | 0.61±0.75 | 0.82±0.71 | <.001** |
| ODI | 0.56±0.74 | 0.84±0.71 | <.001** |
| T90 | 2.07±7.95 | 8.45±15.34 | <.001** |
| Mean SpO_2_ | 95.21±2.35 | 93.24±4.37 | <.001** |
| Arousal | 0.58±0.71 | 0.53±0.61 | .038* |
| Snore | 5.59±4.71 | 4.36±3.65 | <.001** |
| NMP | 0.47±0.50 | 0.48±0.50 | .524 |
| IMP | 0.29±0.46 | 0.30±0.46 | .843 |
| CMP | 0.03±0.16 | 0.01±0.12 | .012* |
| SMP | 0.21±0.41 | 0.21±0.41 | .838 |

*p<0.05, ** p < 0.001. AHI = apnea/hypopnea index; ODI = oxygen desaturation index; T90 = percentage of oxygen saturation under 90;SMP = side mouth puffing; CMP = complete mouth puffing; NMP = non-mouth puffing; IMP = intermit-tent mouth puffing; n = the number of data minutes.

In Table S-2, Seven participants’ AHIs are found to be higher at REM than at NREM, and the other three participants’ AHIs are found vice versa. The former are classified as REM-dependent group, and the latter NREM-dependent group. Further study reveals that the former group’s AHI, ODI, T90, IMP are found to be higher at REM than at NREM and that those of the latter group are found to be vice versa.

**Table S-2.** Two groups’ differences of AHI, oxygen related variables, and MPSs at NREM and REM

|  | REM-dependent group (7) | | | NREM-dependent group (3) | | |
| --- | --- | --- | --- | --- | --- | --- |
|  | NREM  (n=2189) | REM  (n=618) | P-value | NREM  (n=750) | REM  (n=148) | P-value |
| AHI | 0.48±0.74 | 0.87±0.72 | <.001** | 0.98±0.67 | 0.61±0.60 | <.001** |
| ODI | 0.42±0.70 | 0.87±0.72 | <.001** | 0.95±0.71 | 0.72±0.66 | <.001** |
| T90 | 2.28±8.53 | 8.54±15.39 | <.001** | 1.44±5.91 | 8.07±15.15 | <.001** |
| Mean SpO_2_ | 95.00±2.43 | 93.05±4.32 | <.001** | 95.82±1.96 | 94.05±4.49 | <.001** |
| Arousal | 0.46±0.69 | 0.54±0.62 | .018* | 0.91±0.68 | 0.48±0.53 | <.001** |
| Snore | 5.74±5.04 | 4.38±3.71 | <.001** | 5.16±3.53 | 4.29±3.41 | .006* |
| NMP | 0.47±0.50 | 0.43±0.50 | .121 | 0.47±0.50 | 0.69±0.46 | <.001** |
| IMP | 0.25±0.43 | 0.30±0.46 | .012* | 0.43±0.50 | 0.29±0.46 | .001* |
| CMP | 0.04±0.19 | 0.02±0.13 | .007* | 0.01±0.06 | 0 | .441 |
| SMP | 0.25±0.43 | 0.25±0.43 | .953 | 0.09±0.29 | 0.02±0.14 | <.001** |

*p<0.05, ** p < 0.001. AHI = apnea/hypopnea index; ODI = oxygen desaturation index; T90 = percentage of oxygen saturation under 90; SMP = side mouth puffing; CMP = complete mouth puffing; NMP = non-mouth puffing; IMP = intermit-tent mouth puffing; n = the number of data minutes.
